# Automatic mandibular third molar and mandibular canal relationship determination based on deep learning models for preoperative risk reduction

**DOI:** 10.1007/s00784-025-06285-6

**Published:** 2025-03-25

**Authors:** Elham Tahsin Yasin, Mediha Erturk, Melek Tassoker, Murat Koklu

**Affiliations:** 1 Graduate School of Natural and Applied Sciences, Department of Computer Engineering, Faculty of Technology, Selcuk University, Konya, Türkiye; 2 Department of Dentomaxillofacial Radiology, Faculty of Dentistry, Necmettin Erbakan University, Konya, Türkiye; 3 Department of Computer Engineering, Faculty of Technology, Selcuk University, Konya, Türkiye

**Keywords:** Cone beam computed tomography, Deep learning models, Dental imaging, Mandibular canal, Mandibular third molar, Medical image analysis

## Abstract

**Objectives:**

This study explores the application of deep learning models for classifying the spatial relationship between mandibular third molars and the mandibular canal using cone-beam computed tomography images. Accurate classification of this relationship is essential for preoperative planning, as improper assessment can lead to complications such as inferior alveolar nerve injury during extractions.

**Materials and Methods:**

A dataset of 305 cone-beam computed tomography scans, categorized into three classes (not contacted, nearly contacted, and contacted), was meticulously annotated and validated by maxillofacial radiology experts to ensure reliability. Multiple state-of-the-art convolutional neural networks, including MobileNet, Xception, and DenseNet201, were trained and evaluated. Performance metrics were analysed.

**Results:**

MobileNet achieved the highest overall performance, with an accuracy of 99.44%. Xception and DenseNet201 also demonstrated strong classification capabilities, with accuracies of 98.74% and 98.73%, respectively.

**Conclusions:**

These results highlight the potential of deep learning models to automate and improve the accuracy and consistency of mandibular third molars and the mandibular canal relationship classifications.

**Clinical Relevance:**

The integration of such systems into clinical workflows could enhance surgical risk assessments, streamline diagnostics, and reduce reliance on manual analysis, particularly in resource-constrained settings. This study contributes to advancing the use of artificial intelligence in dental imaging, offering a promising avenue for safer and more efficient surgical planning.

## Introduction

The mandibular third molar (MTM), commonly referred to as the wisdom tooth, is a critical anatomical structure often associated with dental extractions due to its high susceptibility to impaction [1]. Impaction of MTMs occurs when the tooth fails to fully erupt into the oral cavity, often due to spatial constraints or abnormal angulations [2]. This condition frequently leads to complications such as pericoronitis, caries in adjacent teeth, and periodontal issues. More critically, impacted MTMs pose a significant surgical risk due to their close anatomical relationship with the mandibular canal (MC) [3], which houses the inferior alveolar nerve (IAN). Mismanagement during surgical extraction can result in nerve damage, manifesting as temporary or permanent numbness, paresthesia, or dysesthesia in the lower lip, chin, and gingiva. Thus, understanding and accurately classifying the spatial relationship between the MTM and the MC is a fundamental step in preoperative planning [4]. Traditionally, the relationship between MTMs and the MC has been assessed using manual classification techniques on radiographic images [5]. Panoramic radiography, a widely used modality in dental diagnostics, provides a two-dimensional (2D) overview of the jaws and associated structures [6]. Radiologists or clinicians analyse specific signs on panoramic radiographs, such as root darkening, interruption of the MC’s cortical outline, and deviation of the canal’s path, to estimate the proximity between the MTM and the MC [7]. While these classifications have been essential in risk assessment, they are inherently limited by inter-observer variability and the two-dimensional nature of panoramic images [8], which may lead to misinterpretations, particularly in cases were anatomical overlap obscures true spatial relationships [9]. Cone Beam Computed Tomography (CBCT) has emerged as the gold standard for evaluating these relationships due to its three-dimensional (3D) imaging capabilities [10]. However, manual analysis of CBCT images remains labour-intensive and subjective, emphasizing the need for more automated and objective methods [11]. Impacted MTMs represent a significant clinical challenge due to their associated risks. The most serious complication arises from their proximity to the MC. During extraction, inadvertent damage to the IAN can result in sensory disturbances, significantly affecting a patient’s quality of life. The risk of nerve injury is compounded by factors such as deep impaction, unfavourable angulation, and direct contact between the MTM roots, and the MC [12]. Studies have reported that IAN injury occurs in 0.44% to 8.4% of cases, with permanent nerve damage being rarer but particularly debilitating. Accurate preoperative risk stratification is therefore crucial to minimizing complications and ensuring safe surgical outcomes [13–15]. Recent advancements in imaging and computational technologies have paved the way for automatic classification systems, which offer several advantages over manual methods. Automated systems can process large volumes of data rapidly and consistently, eliminating inter- and intra-observer variability [16–19]. By integrating algorithms capable of analysing complex spatial relationships, these systems provide objective and reproducible assessments, aiding clinicians in surgical decision-making. Moreover, automatic classification holds particular promise in resource-constrained settings, where access to experienced radiologists may be limited [20–23].

Deep learning, a subset of artificial intelligence (AI), has gained significant traction in medical image analysis [24] due to its ability to process complex patterns in high-dimensional data. Convolutional Neural Networks (CNNs), in particular, have demonstrated remarkable success in segmentation, classification, and detection tasks within radiology. The application of deep learning models to CBCT images of the MTM-MC relationship offers a transformative approach to preoperative risk assessment [25–28]. By training on labeled datasets, these models can learn to identify nuanced features that might escape manual classification, thereby improving diagnostic accuracy and efficiency. Studies have explored various deep learning architectures, such as U-Net for segmentation and ResNet for classification, to automate the analysis of CBCT scans. These models have achieved high accuracy, sensitivity, and specificity in distinguishing contact and non-contact relationships between MTMs and the MC. Additionally, advancements in transfer learning and ensemble modeling have further enhanced the robustness and generalizability of these systems [28, 29]. By automating the classification process, deep learning not only streamline workflows but also democratizes access to high-quality diagnostic tools, reducing reliance on CBCT and experienced radiologists in underserved regions. The intersection of dentistry and AI heralds a new era in the management of impacted MTMs [2, 10]. Deep learning-based systems offer a promising avenue for improving the accuracy, efficiency, and consistency of preoperative risk assessments. By leveraging CBCT imaging and cutting-edge AI models, these systems can provide clinicians with actionable insights, ultimately enhancing patient safety and outcomes. As research continues to refine these models, the integration of automated classification into routine clinical practice represents a significant leap forward in dental care and surgical planning [17–19]. The primary aim of this study is to develop and validate a robust and accurate deep learning-based system for classifying the anatomical relationship between MTM teeth and the MC using CBCT images. Specifically, the classification process categorizes the relationship into three distinct classes: not contacted, nearly contacted, and contacted. This classification is essential for preoperative risk assessment and surgical planning, as the proximity of the MTM to the MC significantly impacts the likelihood of complications such as inferior alveolar nerve (IAN) injury during third molar extractions.

The primary objectives of this study are multifaceted, aiming to address critical aspects of classifying and automating the evaluation of the relationship between MTM roots and the MC. Firstly, the study defines precise classification criteria. The "not contacted" class includes cases with clear separation between the MTM roots and the MC, with no anatomical interaction. The "nearly contacted" class describes cases where the MTM roots are closely approximated to the MC but maintain an intact cortical layer without direct contact. Finally, the "contacted" class encompasses cases where the MTM roots are in direct contact with or invaginate into the MC, posing the highest risk for surgical complications.

To ensure the reliability and accuracy of the dataset, the study uses CBCT images meticulously annotated and classified by three experienced dentists specializing in tomography and maxillofacial radiology. A rigorous double-checking process minimizes observer biases and errors, establishing a gold standard for training and evaluating deep learning models. This manual validation provides a reliable benchmark for automated classifications. The study also focuses on developing and testing state-of-the-art deep learning models, employing architecture such as CNNs for classification tasks. These models aim to automate the classification of MTM-MC relationships, reducing the need for manual analysis and offering rapid, reproducible, and scalable diagnostic tools. Multiple models are evaluated for accuracy, sensitivity, and specificity across diverse patient cases. Addressing the limitations of manual classification is another key objective. While reliable, manual methods are time-consuming and subject to inter- and intra-observer variability. By leveraging deep learning models, the study seeks to standardize the classification process, reducing subjective variability and enhancing diagnostic consistency. This automation also democratizes access to high-quality diagnostic tools, particularly in regions with limited access to specialized radiologists.

To validate the deep learning models, the study tests their performance on a hold-out dataset, assessing their ability to accurately classify MTM-MC relationships. Metrics such as accuracy, precision, recall, and F1 score benchmark the models against expert classifications.

Finally, the study aims to contribute to improved clinical decision-making and patient outcomes. By providing an automated and reliable classification system, it seeks to enhance preoperative evaluations, enabling clinicians to better assess surgical risks and tailor their approaches. This system is designed to complement clinical expertise, supporting safer and more effective surgical interventions. Also to bridge the gap between manual expertise and automated precision by leveraging deep learning models to classify MTM-MC relationships with high reliability and accuracy. The rigorous dataset validation by three expert dentists underscores the study’s commitment to ensuring data quality, while the integration of advanced AI techniques highlights its potential to revolutionize diagnostic workflows in dental and maxillofacial care.

Khorshidi et al. (2024) goaled in the research study to build an AI-based prognosis model for the jaw third molar extraction, which would in turn make surgical planning much more accurate, thus reducing the risks of postoperative complications. The set contains 738 CBCT radiology reports and the reports are based upon determining factors such as the position of the tooth, the number, and the shape, the proximity to the MC, and root curvature. The result is a deep learning model trained on rule-based NLP algorithm after preprocessing and feature extraction of 556 cases and validating on it with 182 cases. The model demonstrated four categories of tooth extraction difficulty with an accuracy of 95% for both datasets. The model precision for both sets of data was at 0.97, while recall was 0.95 and 0.89 for the two sets of data, respectively [30].

Kumbasar et al. (2024) studied the relation between MTM and the alveolar nerve in panoramic radiographs using AI. 544 X-rays were labeled with CBCT was used to develop an AI model to find out the relation of MTM with IAN, thus avoid high radiation from CBCT. Image enhancement was with CLAHE for better visibility. Classification with AlexNet, VGG16, and VVG19 into 4 classes related, lingual, vestibule, and others showed accuracy rates of 94.1%, 80.6%, 74.6%, and 79.7% respectively [31].

Jing et al. (2024) created MM3-IACnet, a new AI tool that recognizes where tooth roots are near the jaw’s nerve canal in Panoramic X-rays. The purpose reduces the need for full-scan CT and solves problems in old scans. They tested it on 1,570 pairs of X-rays and accurate results showed 88.5% [32].

Fang et al. (2024) developed an AI tool to detect two crucial nerves in dental X-rays. This tool can help dentists in planning surgeries while avoiding nerve damage. They used 450 X-ray images, including those from a Chinese hospital and a public platform. The AI tool has a detection accuracy of 92.56% and is 3.06% better than other methods. Thanks to AI-based image enhancement, dentists can now easily see the critical mandibular foramen and canal in panoramic radiographs [33].

Unal and Pekiner (2024) explored the use of deep learning for identifying the MC in relation to the MTM using CBCT images. A dataset of 300 patient CBCT scans, converted from DICOM to JPEG and annotated, was used. The data was split into a training set (n = 270) and a test set (n = 30). Results showed accuracy (99%) and segmentation capabilities, with sensitivity at 75%, precision at 78%, and a Dice score of 0.76 [34].

Barnes et al. (2024) developed AI models to automate the classification of the MC’s spatial relationship to the third molar using CBCT images, aiming to reduce manual effort and improve diagnostic accuracy. A dataset of 434 annotated CBCT scans (ages 18–60) categorized the canal’s position as lingual, buccal, or inferior. Two CNNs, AlexNet and ResNet50, were trained and tested on this dataset, split into 262 training and 172 testing images. ResNet50 outperformed AlexNet, achieving 83% accuracy compared to AlexNet’s 81% [35].

Aung et al. (2024) presented a deep learning approach for automated MC segmentation on panoramic radiographs, overcoming challenges related to manual segmentation and device variability. A dataset of 2,100 radiographs from three imaging devices (RAYSCAN Alpha (PAN A), OP-100 (PAN B), and CS8100 (PAN C)) was annotated by expert oral radiologists and used to train U-Net-based CNNs with backbones including EfficientNetB4, ResNet50, ResNet152, and SEResNet152. Results showed that the multi-device training approach achieving an average DSC of 88.9%, precision of 90.6%, and recall of 87.4%. Single-device-trained models performed worse, underscoring the importance of diverse training data. Among the backbones, EfficientNetB4 demonstrated superior performance in segmentation tasks [36].

Fukuda et al. (2022) developed and evaluated a deep learning (DL) system to predict the 3D contact status between MTM and the MC using panoramic radiographs. A dataset of 800 image patches, evenly split between high-risk and low-risk groups based on CBCT findings, was used. The data was divided into 700 training and 100 testing patches, with augmentation techniques applied to improve model performance. The GoogLeNet architecture was trained over 100 epochs using stochastic gradient descent. The DL model achieved the highest AUC (0.85), surpassing residents (0.55) and matching radiologists (0.81). It also demonstrated superior sensitivity and specificity, with an interclass correlation coefficients (ICC) of 0.69, indicating higher diagnostic reproducibility compared to radiologists (0.54) and residents (0.19) [37].

Liu et al. (2022) developed a deep learning system to automate the detection and classification of relationships between MTM and the MC on CBCT images, aiming to assist clinicians in preoperative risk assessment and reduce the risk of inferior alveolar nerve (IAN) injury. The dataset included 254 CBCT scans of patients aged 15–64, annotated by radiologists to classify MTM-MC relationships into three types: Type I (separation with cancellous bone), Type II (contact with an intact cortical border), and Type III (invagination with cortical border interruption). A two-step workflow employed U-Net for segmentation and ResNet-34 for classification. Performance was evaluated using Dice Similarity Coefficient (DSC), Intersection over Union (IoU), and classification metrics. MTM segmentation achieved a DSC of 0.973 and IoU of 0.961, while MC segmentation achieved a DSC of 0.925 and IoU of 0.900. The combined model achieved a classification accuracy of 93.3%, with sensitivity of 90.2% and specificity of 95.0%, performing comparably to expert radiologists, particularly in detecting higher-risk Types II and III relationships [38].

## Material and methods

**Fig. 1:**
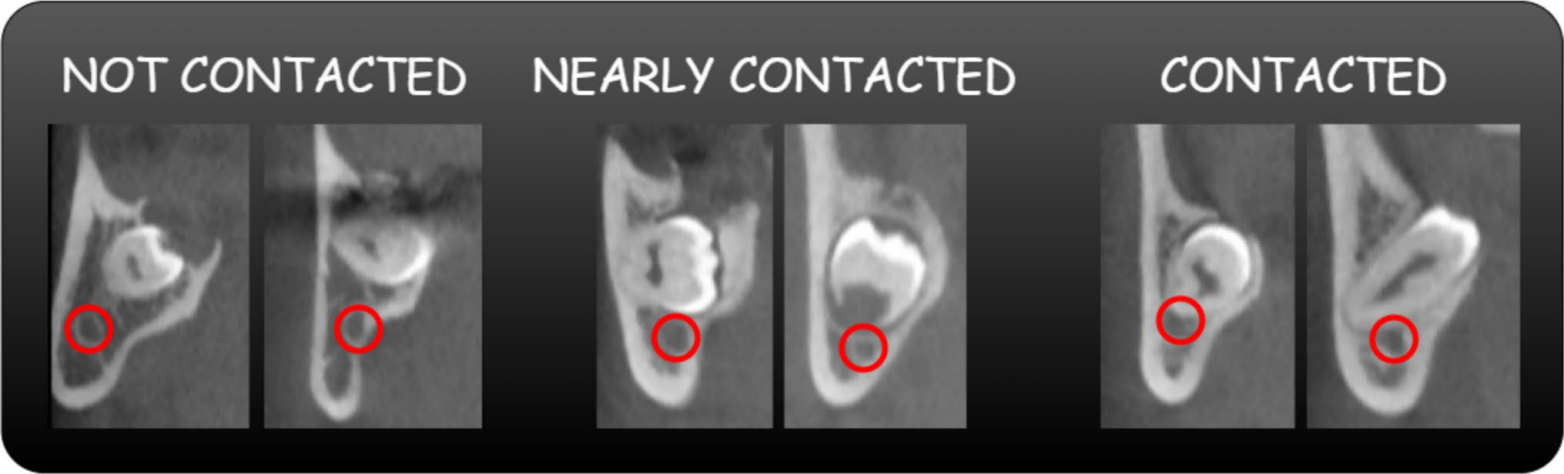
Classes image representing the relationship between MTM and MC

**Fig. 2:**
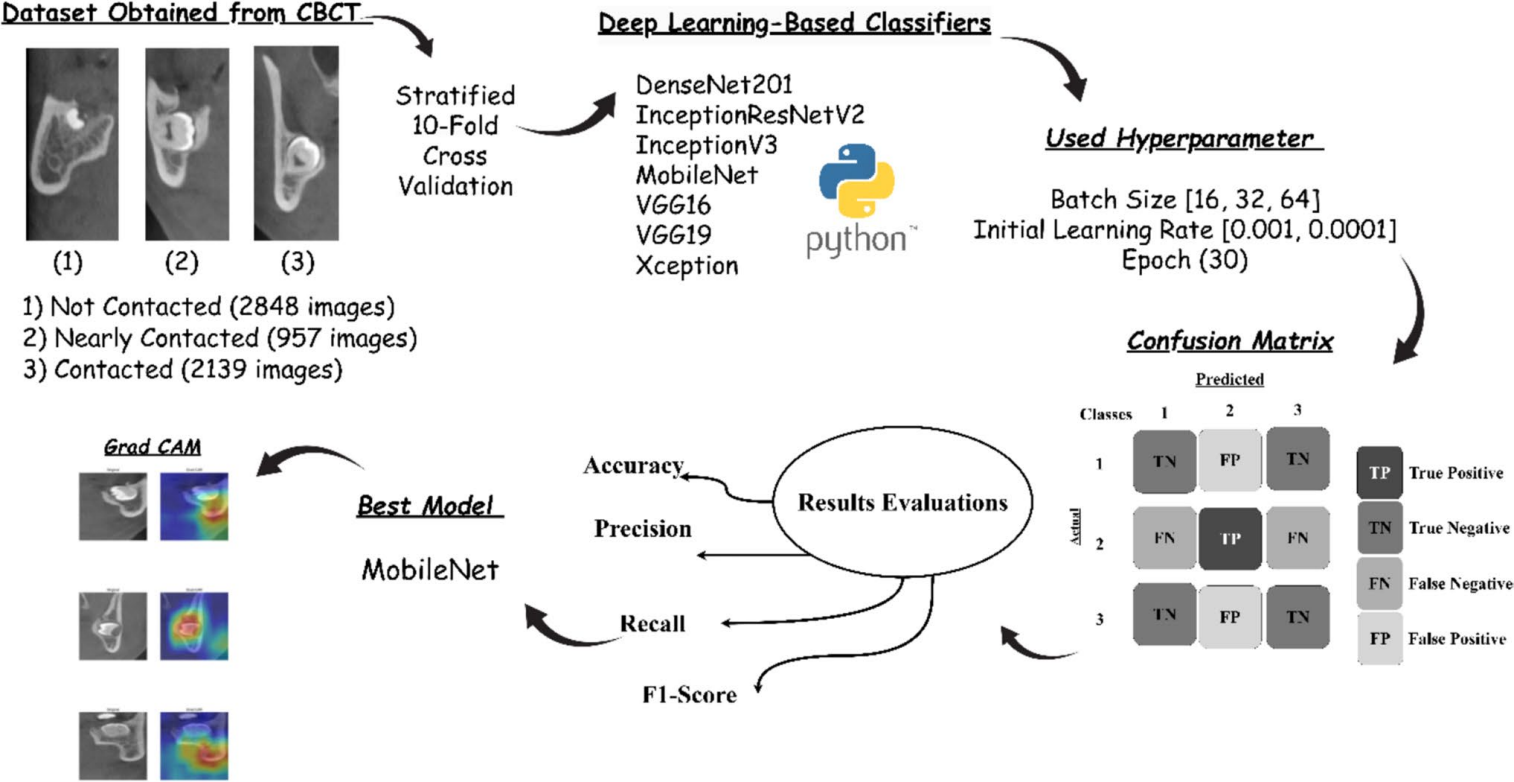
Mandibular third molar and mandibular canal relationship classification flow diagram

This study used a dataset comprising 305 patient’s CBCT, categorized into three classes based on the anatomical relationship between mandibular third molars (MTMs) and the MC: not contacted (clear separation with no interaction), nearly contacted (close approximation with an intact cortical layer), and contacted (direct contact or invagination into the MC) (Fig. 1) [38, 39]. The images were manually annotated and double-checked by three experienced dentists specializing in maxillofacial radiology from Necmettin Erbakan University to ensure the accuracy and consistency of classifications. CBCT images were obtained using the Morita 3D Accuitomo 170 (J Morita MFG Corp., Kyoto, Japan) 3D imaging system. The dataset was divided into training and testing subsets by stratified cross validation with tenfold for the deep learning experiments. The dataset was divided into 10 equal parts for cross-validation, where the model was trained on 9 folds and tested on the remaining fold in each iteration. This process was repeated 10 times, with the final performance metrics averaged across all iterations for reliable evaluation. A flow diagram representing the study process is presented in Fig. 2.

To classify these relationships, various deep learning architectures were employed, including DenseNet201 [40, 41], InceptionResNetV2 [42–45], InceptionV3 [43, 46, 47], MobileNet [48, 49], VGG16 [50, 51], VGG19 [50, 52], and Xception [53]. These models were selected due to their proven effectiveness in analysing complex spatial patterns in medical imaging tasks. Each model was trained with varying configurations to optimize performance, including batch sizes of 16, 32, and 64, learning rates of 0.001 and 0.0001 [54], and 30 epochs. The Adam optimizer and categorical cross-entropy loss function were used during training.  

The models were evaluated using several performance metrics, including accuracy [55, 56], precision, recall, F1-score [57], log-loss, Cohen’s kappa, and the area under the receiver operating characteristic curve (ROC-AUC). Accuracy measured overall classification correctness, while precision and recall evaluated the model’s ability to correctly classify positive cases. The F1-score provided a balance between precision and recall, and log loss assessed the reliability of predicted probabilities. Cohen’s Kappa (κ) is a statistical measure of inter-rater agreement based on the fact that agreement could occur by chance. ROC AUC was used to gauge the models’ discriminatory capabilities across different thresholds.

Each model was trained with all combinations of batch sizes and learning rates to identify the optimal configuration. After training, the models’ performance was analysed, and comparative evaluations were conducted to assess the impact of different batch sizes and learning rates on classification results. ROC curves [58–61] were plotted to visualize the models’ ability to differentiate between the three classes.

This methodology was implemented using Python programming, leveraging deep learning frameworks such as TensorFlow and Keras [53, 62]. Data analysis and visualization were performed using libraries like NumPy, pandas, and Matplotlib. By systematically training and evaluating multiple deep learning architectures, this study aimed to identify the optimal model and parameter settings for accurately classifying MTM-MC relationships, ultimately contributing to improved clinical decision-making and surgical safety.

### Dataset

**Fig. 3:**
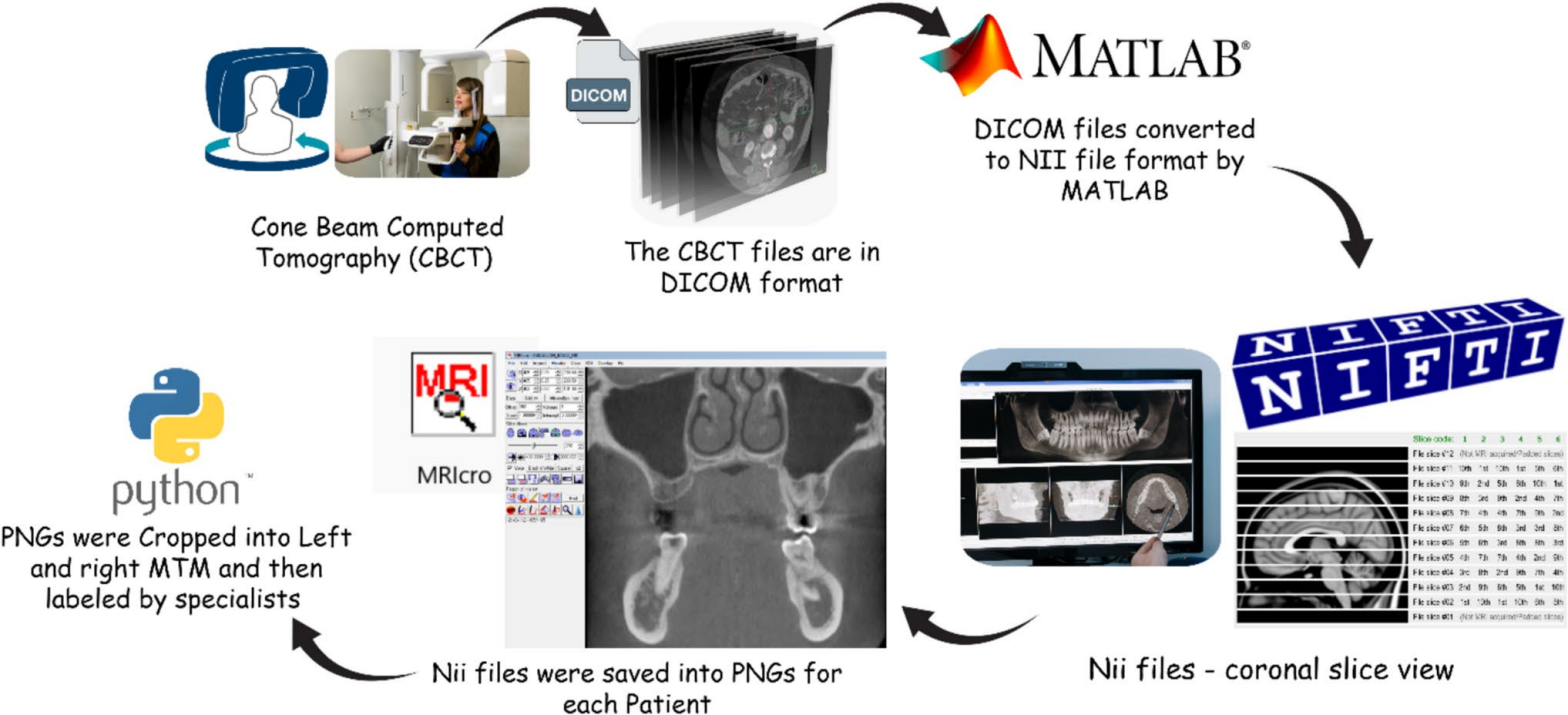
Conversion of cone beam computed tomography to PNG process steps

According to the anatomical relationship between the MTMs and the MC, 305 patient CBCTs (parasagittal slices) were classified into three classes: 2848.PNG images were not contacted (clear separation with no interaction), 957.PNG images were nearly contacted (close approximation with intact cortical layers), and 2139.PNG images were contacted (direct contact or invagination into the MC). To ensure accuracy and consistency of classifications, the images were manually annotated and double-checked by three dentists specializing in maxillofacial radiology at Necmettin Erbakan University. An ethics committee approval was obtained from the Department of Dentomaxillofacial Radiology at Necmettin Erbakan University, Konya, Turkey. Cross validation [63] method was selected for data split. In each iteration, the model was trained on 9 folds and tested on the remaining fold, dividing the dataset into 10 equal parts. The process was repeated 10 times, with the final performance metrics being averaged across all iterations. Cross-validation, which is a widespread method in evaluating machine learning models, can be described as the process of breaking down a dataset into a training and validation set and then analysing the difference in the performance in separate steps. The traditional k-fold cross-validation method divides the dataset into k equal parts (folds) and trains the model k times; each time a different fold function is used for the validation set while the remaining k-1 folds are used for training. Nevertheless, this method does not account for the class distribution patterns, and as a result, the folds may present an imbalance, particularly when working with unbalanced data sets. Stratified k-fold cross-validation, on the other hand, makes sure that every fold keeps the same class ratio as the original dataset. It is especially essential for classification tasks that involve the classes with fewer samples because it stops the model from being biased toward the majority class and provides a more reliable estimate of performance. Stratified cross-validation is usually the best choice in comparison to the usual k-fold cross-validation, especially to classification problems, where it makes sure that all classes are represented evenly in every iteration [64, 65]. Figure 3 illustrates the steps for creating the dataset.

**Fig. 4:**
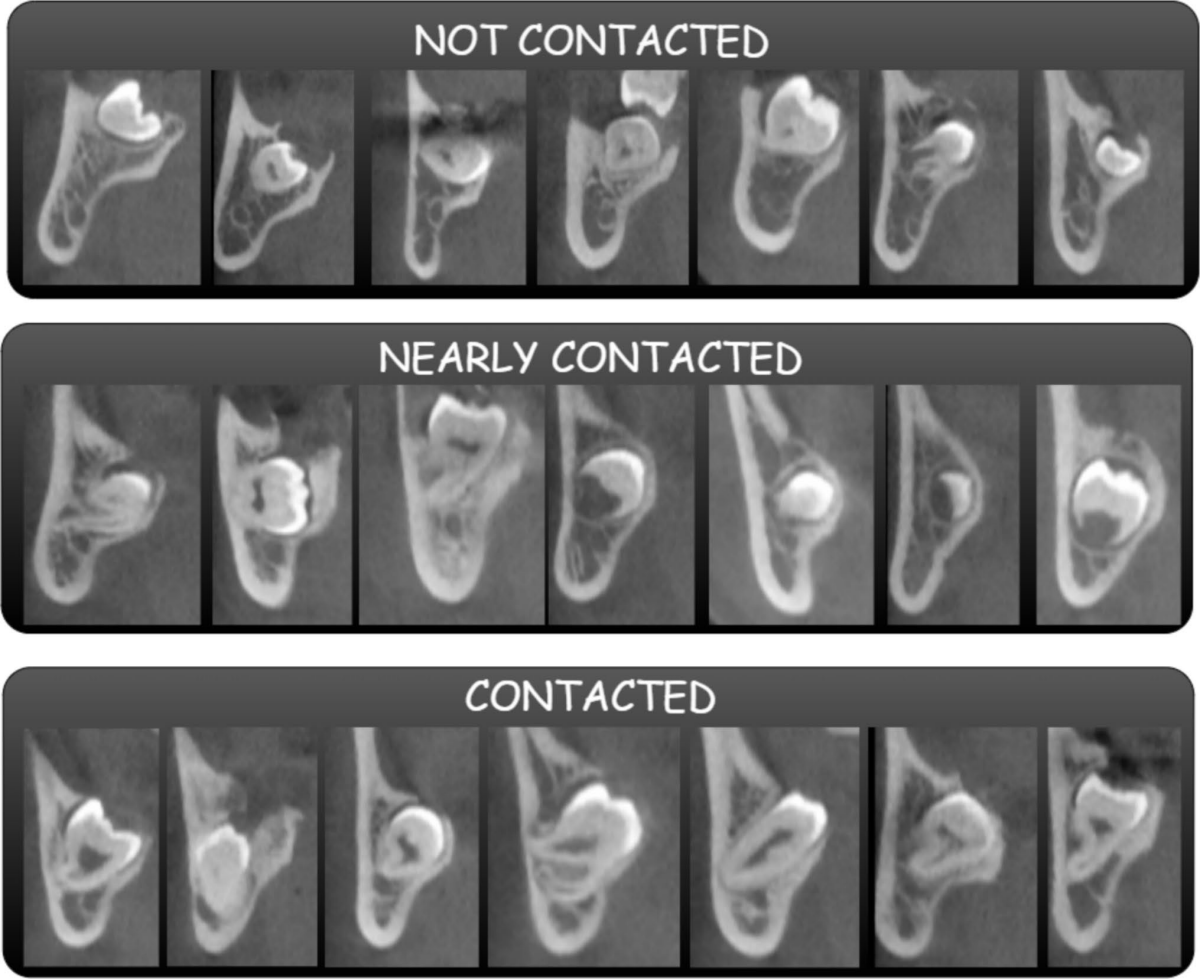
Sample images presentation of MTM-MC dataset

MTMs and MCs are categorized into three groups based on their anatomical relationship. There are three types of contact: not contacted, nearly contacted, and contacted. Figure 4 shows sample images from the dataset.

The initial CBCT scans were stored in DICOM format. This method was created to guarantee that all the images were done in a way that makes it easy for anyone to use deep learning for the assessment of the images. The files were later turned into NIfTI (nii) format via a MATLAB-based script that enabled better control of the image dimensions and a consistent coronal view. The MRIcro software (v1.40 build 1) was also used to extract PNG images from each NIfTI file. The program developed a bone contrast preset (400:2000, brightness: contrast) that was applied to enhance the visibility of anatomical structures. Because the maxillary third molars (MTM) were particularly visible on both (left and right) sides during the first round, the Python programming language was used to perform the image cropping preprocess. The images were cropped as left and right MTM. This pre-processing pipeline is a systematic process that converts clinical CBCT scans into deep learning-compatible image file formats thus preserving anatomical integrity and allowing for reproducibility. The time required for converting DICOM images to NII format, saving the NII files as PNGs, and cropping the images into left and right sections was relatively short. However, the annotation process by specialists was time-consuming since they performed manual labelling. Apart from annotation, the preprocessing steps for a single patient’s images took approximately 45 s (as shown in Fig. 3).

### Deep learning models

The process of deep learning uses artificial neural networks to learn from data. Typically, deep learning algorithms are trained on large datasets of labelled data. Data features are associated with correct labels by the algorithms. Deep learning algorithms [66, 67] can make predictions on new data once they have been trained. To identify teeth in new images, a deep learning algorithm that has been trained to recognize teeth can be used. Deep CNNs [68–70] are widely used in computer vision tasks such as image classification [71], such as DenseNet201 [40, 41], InceptionResNetV2 [42–45], InceptionV3 [43, 46, 47], MobileNet [48, 49], VGG16 [50, 51], VGG19 [50, 52], and Xception [53]. Various models were developed to address different challenges or improve various aspects of performance, such as efficiency, accuracy, or computational cost.

Each model architecture has unique design characteristics that target specific needs. For example, DenseNet201 emphasizes efficient feature reuse by connecting each layer to every other layer, improving gradient flow and reducing the number of parameters. InceptionResNetV2 and InceptionV3 integrate the strengths of inception modules for capturing multi-scale features with computational efficiency. MobileNet is optimized for mobile and embedded devices, achieving high accuracy with lower computational cost, while VGG16 and VGG19 are known for their simplicity and deeper architectures, which stack small convolutional filters for hierarchical feature learning. Xception leverages depthwise separable convolutions to enhance both performance and efficiency. Selecting a suitable model depends on the specific application requirements and constraints. Parameter for each model presented in Table 1.

**Table 1:** Parameter and layer number details for each model

Model	Parameters (millions)	Number of Layers
DenseNet201	20.0	201
InceptionResNetV2	55.9	164
InceptionV3	23.9	159
MobileNet	4.2	88
VGG16	138.4	16
VGG19	143.7	19
Xception	22.9	71

Table 1 represents the total number of trainable parameters and the number of layers within the network. Layers refer to the depth of a model, excluding input and output layers in some cases.

### Classification evaluation and confusion matrix

A classification model’s performance must be evaluated to ensure that it is accurate and effective. In addition to accuracy, there are other factors to consider. Several other metrics [72–75] can be used to assess the performance of your model. Based on input data, classification metrics predict class labels. There are only two possible output classes in binary classification. Multiclass classification involves more than two possible classes. In Cohen’s Kappa, ${P}_{o}$​ (Observed Agreement) which is the proportion of instances that the true labels and the predicted labels are found to coincide in. ${P}_{e}$ (Expected Agreement) which is the agreement to be expected due to randomness, and which is calculated according to the proportions of the classes in both the true and the predicted labels. Formula [63, 76, 77] for each metric is presented in Table 2.

**Table 2:** Presentation of evaluation metrics formulas

Metrics	Formulas
Accuracy	$Accuracy=\frac{TP+TN}{TP+TN+FP+FN}$
Precision	$Precision= \frac{TP}{TP+FP}$
Recall	$Recall= \frac{TP}{TP+FN}$
F1-Score	$F1-Score= 2 \times \frac{Precision \times Recall}{Precision+Recall}$
Cohen’s Kappa	$k= \frac{{P}_{o}-{P}_{e}}{1 - {P}_{e}}$

**Fig. 5:**
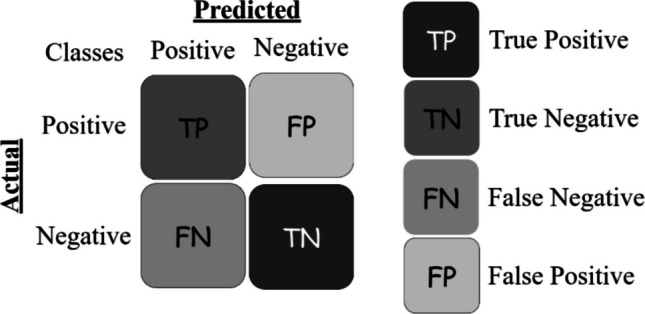
Binary confusion matrix

**Fig. 6:**
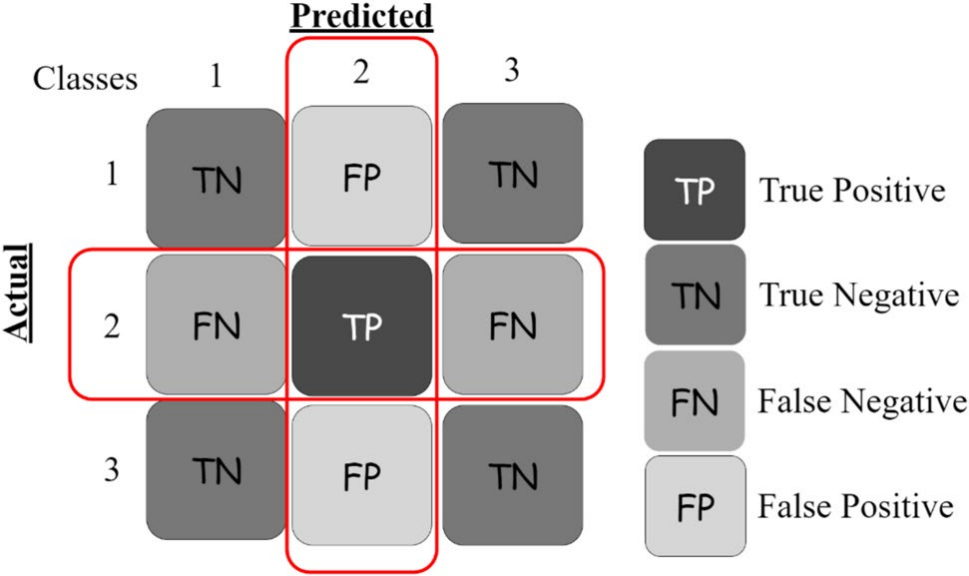
Multiclass confusion matrix (3 classed)

In classification tasks, confusion matrices are essential for evaluating model performance, providing detailed insights into the model’s predictions. For binary classification, the confusion matrix [78, 79] consists of four key components: true positives (TP), true negatives (TN), false positives (FP), and false negatives (FN), which collectively summarize the model’s predictions for two possible classes, shown in Fig. 5. In contrast, multiclass confusion matrices extend this concept to multiple classes, such as a three-class problem where the matrix becomes a 3 × 3 grid. Each cell in the matrix represents the count of instances where the true class corresponds to a particular row and the predicted class corresponds to a column. For example, in a three-class problem (e.g., Class 1, Class 2, Class 3) [80] (Fig. 6), the diagonal entries indicate correctly classified instances for each class, while off-diagonal entries show misclassifications. This detailed breakdown helps identify performance disparities across classes, guiding improvements in model training and addressing class imbalances [81, 82].

### Grad-CAM and its role in deep learning analysis

Gradient-weighted Class Activation Mapping (Grad-CAM) [83, 84] is a visualization technique widely used to interpret the decisions made by CNNs. It provides a way to understand which regions in an input image are most influential in the model’s decision-making process. Grad-CAM achieves this by leveraging the gradients flowing back from a specific layer, often the final convolutional layer, to compute a heatmap. This heatmap highlights areas of the input image that contribute most significantly to a specific prediction, offering insights into the inner workings of the network [85–87].

## Experimental results

Models trained for the MTM dataset, and the obtained results presented in this section. The performance of multiple deep learning models (DenseNet201, InceptionResNetV2, InceptionV3, MobileNet, VGG16, VGG19, and Xception) was evaluated for classifying the relationship between MTMs and the MC. These models were tested with two learning rates (0.001 and 0.0001), batch size of (16, 32, and 64) and 30 epochs. All experiments were conducted on a system equipped with a 12th Gen Intel® Core™ i7-12650H processor (2.30 GHz) and 32 GB of RAM. The system operated on a 64-bit Windows OS with × 64-based processor architecture. GPU-accelerated training was performed using CUDA 11.2 (release 11.2, V11.2.67) to leverage hardware acceleration for deep learning computations. The GPU memory allocation was dynamically managed by TensorFlow to optimize performance. To ensure computational reproducibility, we monitored per-epoch training times, which varied based on model complexity, batch size, and learning rate. Metrics such as accuracy, precision, recall, F1-score, log-loss, Cohen’s Kappa, and ROC-AUC were used for evaluation.

Based on the provided Table 3, MobileNet consistently achieved the highest accuracy, precision, recall, and F1-score, particularly with a learning rate of 0.0001, where it reached an accuracy of 99.44%, precision of 99.45%, recall of 99.44%, and an F1-score of 99.44%. The log loss was also minimal (0.022), indicating its strong predictive capability. The ROC-AUC value of 0.9993 underscores its exceptional discrimination ability.

**Table 3:** Pre-trained deep learning models obtained results based on 16 batch size

Model	Batch	Learning Rate	Accuracy	Precision	Recall	F1-Score	Log_Loss	ROC-AUC	Cohen’s Kappa
DenseNet201	16	0.001	0.987381	0.987457	0.987381	0.98737	0.052156	0.998774	0.9862
		0.0001	0.987046	0.987119	0.987046	0.987032	0.04871	0.99862	0.9889
InceptionResNetV2		0.001	0.918585	0.924093	0.918585	0.918368	0.240885	0.987038	0.8707
		0.0001	0.925637	0.927865	0.925637	0.925503	0.217854	0.987537	0.8868
InceptionV3		0.001	0.966352	0.966508	0.966352	0.966267	0.134967	0.995444	0.9503
		0.0001	0.974596	0.974625	0.974596	0.974536	0.08695	0.996876	0.9695
MobileNet		0.001	0.993439	0.993473	0.993439	0.993433	0.026019	0.999341	0.9972
		**0.0001**	**0.994448**	**0.994496**	**0.994448**	**0.994442**	**0.02227**	**0.999357**	**0.9972**
VGG16		0.001	0.909994	0.91587	0.909994	0.908447	0.232855	0.98811	0.8586
		0.0001	0.780283	0.786408	0.780283	0.773085	0.561598	0.909916	0.6381
VGG19		0.001	0.858842	0.868057	0.858842	0.857177	0.383995	0.962775	0.7751
		0.0001	0.730643	0.741335	0.730643	0.712119	0.65418	0.879871	0.5436
Xception		0.001	0.987381	0.987448	0.987381	0.987354	0.05183	0.998939	0.9862
		0.0001	0.984859	0.985055	0.984859	0.984809	0.05405	0.998579	0.9834

Xception also performed very well, achieving an accuracy of 98.74% with a learning rate of 0.001. Its precision, recall, and F1-score were all approximately 98.74%, with a log loss of 0.0518 and an impressive ROC-AUC value of 0.9989, demonstrating reliable classification performance.

DenseNet201 showed comparable results, particularly with a learning rate of 0.001, achieving an accuracy of 98.73% and a similar performance for other metrics. This model demonstrated minimal log loss (0.052) and high ROC-AUC (0.9987).

**Fig. 7:**
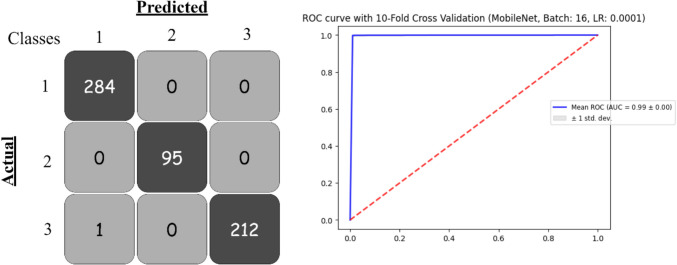
Confusion matrix and ROC curve for best model with 16 batch size

InceptionV3 achieved accuracy in the mid-90s. With a learning rate of 0.0001, its accuracy increased to 97.45%, with precision, recall, and F1-score following suit. It had a relatively low log-loss (0.0869) and a strong ROC-AUC of 0.9968, making it a dependable model for classification tasks. Figure 7 shows the confusion matrix and the ROC curve for the best model.

InceptionResNetV2 showed slightly lower performance compared to InceptionV3, with accuracy values between 91.85% and 92.56%. The log-loss was notably higher than other models (up to 0.2408), indicating less precise predictions, although the ROC-AUC values remained strong (0.9870–0.9875).

VGG16 and VGG19 demonstrated moderate to low performance. VGG16 reached an accuracy of 90.99% with a learning rate of 0.001 but dropped significantly with a lower learning rate. Similarly, VGG19 peaked at 85.88% accuracy with a learning rate of 0.001 but underperformed with lower rates.

**Fig. 8:**
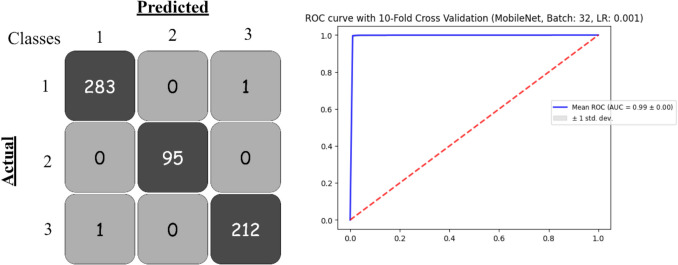
Confusion matrix and ROC curve for best model with 32 batch size

Based on the provided Table 4, the performance of deep learning models (DenseNet201, InceptionResNetV2, InceptionV3, MobileNet, VGG16, VGG19, and Xception) was evaluated with a batch size of 32, using learning rates of 0.001 and 0.0001. Figure 8 presents the confusion matrix and the ROC curve for the best model.

**Table 4:** Pre-trained deep learning models obtained results based on 32 batch size

Model	Batch	Learning Rate	Accuracy	Precision	Recall	F1-Score	Log_Loss	ROC-AUC	Cohen’s Kappa
DenseNet201	32	0.001	0.984521	0.984657	0.984521	0.984495	0.061212	0.998167	0.9862
		0.0001	0.987719	0.987786	0.987719	0.987705	0.047346	0.998797	0.9834
InceptionResNetV2		0.001	0.918731	0.923986	0.918731	0.918609	0.230865	0.987032	0.8708
		0.0001	0.905952	0.909437	0.905952	0.905045	0.278575	0.981167	0.8548
InceptionV3		0.001	0.962818	0.963208	0.962818	0.962833	0.126723	0.995118	0.9476
		0.0001	0.971399	0.971519	0.971399	0.971315	0.097243	0.996155	0.9614
MobileNet		**0.001**	**0.99344**	**0.993476**	**0.99344**	**0.993432**	**0.023606**	**0.999385**	**0.9945**
		0.0001	0.991756	0.991832	0.991756	0.991746	0.031165	0.999364	0.9945
VGG16		0.001	0.905114	0.908884	0.905114	0.904798	0.268624	0.981229	0.8506
		0.0001	0.760764	0.764569	0.760764	0.748726	0.617349	0.887486	0.6033
VGG19		0.001	0.841182	0.849647	0.841182	0.839731	0.409018	0.957151	0.7446
		0.0001	0.696506	0.706705	0.696506	0.678956	0.723522	0.849026	0.4927
Xception		0.001	0.986204	0.986361	0.986204	0.98617	0.050822	0.998743	0.9834
		0.0001	0.980484	0.980739	0.980484	0.980407	0.069286	0.998218	0.9752

Top-performing models demonstrated exceptional results among these, MobileNet consistently outperformed others across both learning rates. At a learning rate of 0.001, it achieved an accuracy of 99.34%, precision of 99.35%, recall of 99.34%, F1-score of 99.34%, log loss of 0.0236, and an impressive ROC-AUC of 0.9994. Even with a reduced learning rate of 0.0001, MobileNet exhibited minimal performance variation, underscoring its robustness. Similarly, Xception produced outstanding results, particularly with a learning rate of 0.001, where it achieved an accuracy of 98.62%, precision of 98.64%, recall of 98.62%, F1-score of 98.62%, log loss of 0.0508, and a ROC-AUC of 0.9987. Like MobileNet, Xception maintained consistent performance at lower learning rates. DenseNet201 also performed reliably, achieving an accuracy of 98.45% at a learning rate of 0.001 and an improved 98.77% at 0.0001. It recorded low log-loss values (0.0612 and 0.0473 for the respective learning rates) and high ROC-AUC values ranging from 0.9981 to 0.9987.

Moderate-performing models included InceptionV3, which excelled with a learning rate of 0.0001, achieving an accuracy of 97.14%, precision of 97.15%, recall of 97.14%, F1-score of 97.13%, log loss of 0.0972, and a ROC-AUC of 0.9962. InceptionResNetV2 showed moderate performance, with accuracy peaking at 91.87% for a learning rate of 0.001. However, its relatively high log-loss values (ranging from 0.2308 to 0.2785) suggested potential weaknesses in predictive probabilities. VGG16 performed well at a learning rate of 0.001, achieving an accuracy of 90.51% and a ROC-AUC of 0.9812, though its performance dropped significantly at lower learning rates.

Underperforming models included VGG19, which achieved a peak accuracy of 84.11% at a learning rate of 0.001 but experienced a significant decline to 69.65% when the learning rate was reduced to 0.0001. Its log loss values were relatively high, particularly at the lower learning rate, indicating suboptimal predictive performance.

**Fig. 9:**
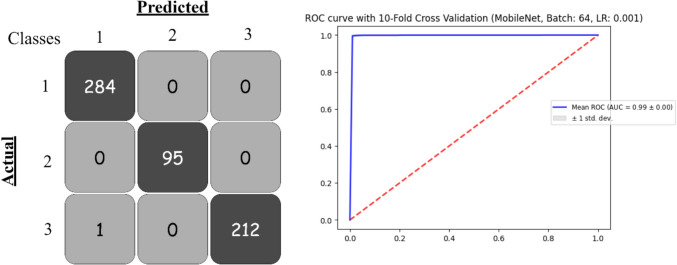
Confusion matrix and ROC curve for best model with 64 batch size

Table 5 provides performance analysis for the batch size 64 for all classifier models. MobileNet emerged as the top-performing model, showcasing remarkable results across both learning rates. At a learning rate of 0.001, it achieved an accuracy of 99.24%, precision of 99.25%, recall of 99.24%, F1-score of 99.24%, a log loss of 0.0258, and an exceptional ROC-AUC of 0.9993. Even with a reduced learning rate of 0.0001, MobileNet maintained strong performance, recording an accuracy of 98.96% and a minimal log loss of 0.0463. DenseNet201 also performed exceptionally well, achieving an accuracy of 98.67% and a log loss of 0.0495 at a learning rate of 0.001, accompanied by a near-perfect ROC-AUC of 0.9986. When the learning rate was lowered to 0.0001, DenseNet201’s accuracy slightly improved to 97.91%, though its log loss increased to 0.0881. Similarly, Xception demonstrated consistent and high performance, achieving its best results at a learning rate of 0.001 with an accuracy of 98.45%, precision of 98.47%, recall of 98.45%, F1-score of 98.45%, log loss of 0.0533, and a ROC-AUC of 0.9986. The ROC curve and confusion matrix for the best model shown in Fig. 9.

**Table 5:** Pre-trained deep learning models obtained results based on 64 batch size

Model	Batch	Learning Rate	Accuracy	Precision	Recall	F1-Score	Log_Loss	ROC-AUC	Cohen’s Kappa
DenseNet201	64	0.001	0.986709	0.986813	0.986709	0.986691	0.049551	0.998612	0.9835
		0.0001	0.979138	0.979306	0.979138	0.979109	0.088142	0.99761	0.9723
InceptionResNetV2		0.001	0.92008	0.923111	0.92008	0.919514	0.219135	0.987039	0.8752
		0.0001	0.8856	0.88774	0.8856	0.8844	0.336157	0.972346	0.8157
InceptionV3		0.001	0.962147	0.962589	0.962147	0.962049	0.12138	0.994964	0.9447
		0.0001	0.963489	0.963813	0.963489	0.963379	0.115156	0.995416	0.9448
MobileNet		**0.001**	**0.99243**	**0.99248**	**0.99243**	**0.99242**	**0.025854**	**0.99934**	**0.9972**
		0.0001	0.98957	0.989619	0.98957	0.989553	0.046308	0.999118	0.9890
VGG16		0.001	0.864565	0.869544	0.864565	0.86309	0.363115	0.963377	0.7835
		0.0001	0.729309	0.728951	0.729309	0.716118	0.678902	0.861231	0.5452
VGG19		0.001	0.811572	0.828788	0.811572	0.804877	0.476276	0.946311	0.6958
		0.0001	0.660161	0.666134	0.660161	0.622713	0.772248	0.815903	0.4115
Xception		0.001	0.984521	0.984758	0.984521	0.984484	0.053342	0.998672	0.9834
		0.0001	0.976782	0.977102	0.976782	0.976682	0.094937	0.997507	0.9723

InceptionV3 performed moderately well, achieving an accuracy of 96.21% at a learning rate of 0.001, with a relatively low log-loss of 0.1213 and a ROC-AUC of 0.9949. At a lower learning rate, its accuracy improved slightly to 96.34%, along with a higher ROC-AUC of 0.9954. InceptionResNetV2 showed moderate performance, with its best accuracy reaching 92.00% at a learning rate of 0.001. However, its relatively high log-loss values (ranging from 0.2191 to 0.3361) suggested less precise predictability. VGG16 demonstrated decent performance at a learning rate of 0.001, achieving an accuracy of 86.45% and a higher log-loss of 0.3631. However, its performance dropped significantly at lower learning rates, with accuracy falling to 72.93%.

VGG19 was among the underperforming models, with its accuracy peaking at 81.15% at a learning rate of 0.001. However, its performance dropped significantly to 66.01% when the learning rate was lowered to 0.0001. Additionally, VGG19 exhibited high log-loss values, reaching up to 0.7722, reflecting challenges in prediction precision.

## Discussion

Recent advancements in dental research have predominantly centered on age prediction and segmentation tasks, with a notable emphasis on employing CNNs for these purposes. Studies frequently utilize architecture such as U-Net and various versions of YOLO for segmentation tasks. For instance, a study developed a U-Net model for early dental caries detection in bitewing radiographs, demonstrating significant improvements in diagnostic performance [88–90]. Another research employed a YOLO-V5-based deep learning approach for tooth detection and segmentation on pediatric panoramic radiographs, achieving satisfied precision and recall rates [91, 92].

While panoramic radiographs have been the primary focus in many studies, there is a growing interest in using CBCT for dental imaging due to its ability to produce detailed, three-dimensional images of dental structures. Recent reviews highlight the application of AI techniques, including deep learning, in CBCT imaging for tasks such as lesion detection and classification [88, 93, 94]

In contrast to the prevalent focus on segmentation, classification tasks in dental imaging have received comparatively less attention. However, there is a trend towards employing pre-trained deep learning models for classification purposes, with efforts to optimize hyperparameters to achieve superior results. For example, an enhanced ResNet50 architecture integrated with a spatial attention mechanism has been proposed to improve classification accuracy in dental diagnostics [95–97]. While segmentation tasks have dominated dental imaging research, there is a discernible shift towards classification, particularly using advanced deep learning models and CBCT imaging, to enhance diagnostic accuracy and efficiency [98, 99].

While discussing this study, the models generally performed better with a learning rate of 0.0001 compared to 0.001. This effect was particularly evident in MobileNet, DenseNet201, and Xception, where lower learning rates yielded higher accuracy, precision, recall, and F1-scores. Lower learning rates likely allowed the models to converge more effectively, avoiding overshooting optimal weights. All models were evaluated with different batch sizes of 16, 32, and 64. The batch size of 16 provided sufficient stability for gradient updates while allowing for efficient training. Further experiments with different batch sizes could offer insights into optimal configurations for these models. Accuracy and F1-Score for MobileNet, Xception, and DenseNet201 consistently excelled across these metrics, showing their ability to balance precision and recall effectively. The best-performing models, including MobileNet and DenseNet201, demonstrated minimal log loss, reflecting confident predictions with high probability values.

A learning rate of 0.0001 generally led to improved performance for most models, particularly DenseNet201 and Xception. However, MobileNet exhibited consistent performance across both learning rates, indicating stability and adaptability. Using a batch size of 32 yielded strong results for lightweight models like MobileNet and DenseNet201, which managed efficient gradient updates and convergence.

Models generally performed better with a learning rate of 0.001. MobileNet, DenseNet201, and Xception maintained excellent performance across both learning rates, while others, like VGG16 and VGG19, showed significant declines with lower learning rates. A batch size of 64 worked well for lightweight models like MobileNet and Xception, as well as DenseNet201, which efficiently utilized this configuration for consistent convergence and stability. MobileNet continues to stand out as the most reliable model across all configurations, delivering exceptional accuracy, minimal log-loss, and near-perfect ROC-AUC. DenseNet201 and Xception also performed consistently well, proving to be viable alternatives for automatic classification tasks. In contrast, VGG19 require further optimization to achieve competitive results. A learning rate of 0.001 generally worked better for most models, particularly when paired with a batch size of 64. These findings reinforce the suitability of lightweight models and advanced architectures for classifying MTM relationships with high precision and reliability.

In this study, Grad-CAM has been applied to visualize and analyze the spatial relationship between MTMs and the MC. The technique serves two primary purposes; Interpretability, By superimposing Grad-CAM heatmaps onto the original CBCT images, clinicians can identify the regions that the deep learning models deemed important for classification. This enhances trust in automated systems by making their predictions more transparent. Secondly Model Validation, Grad-CAM can be used to validate whether the model (MobileNet) is focusing on clinically relevant areas, such as the contact points between MTM roots and the MC. If the highlighted regions align with expert knowledge, it strengthens confidence in the model’s robustness.

**Fig. 10:**
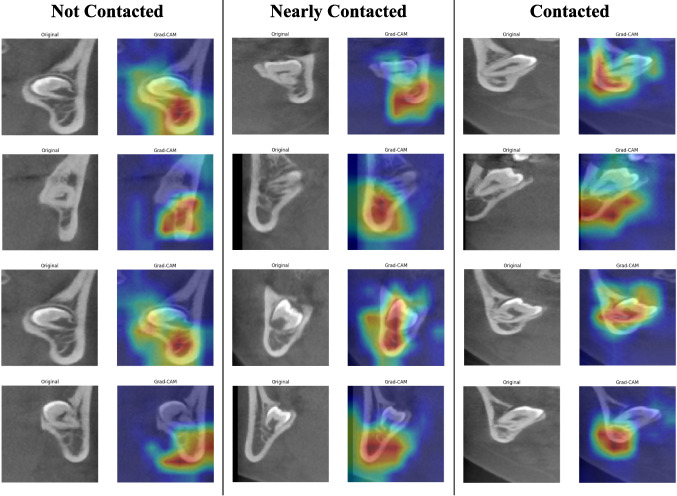
Application for Grad-CAM in MTM-MC relationship

The provided Grad-CAM visualizations in Fig. 10 show the model’s focus on critical areas in CBCT images, highlighting its potential to assist clinicians in preoperative evaluations. The accompanying heatmaps illustrate that the deep learning models successfully prioritize regions that correspond to anatomical features crucial for accurate classification. The specialists conducted a thorough review of the results, ensuring their accuracy was indeed accurate.

The Grad-CAM heatmaps provided in this study demonstrate the models’ capability to correctly identify critical regions associated with MTM-MC relationships. For instance:

In the "not contacted" class, the heatmaps show minimal focus on the MC region, aligning with the absence of direct interaction between the MTM roots and the MC.

For the "nearly contacted" class, the highlighted areas are concentrated near the cortical boundary, reflecting the close approximation without direct contact.

In the "contacted" class, the heatmaps reveal intense focus on the overlapping regions between the MTM roots and the MC, consistent with the highest risk category.

## Conclusion

This study demonstrates the efficacy of deep learning models in classifying the spatial relationships between MTMs and the MC using CBCT images. Among the tested models, MobileNet consistently achieved the highest performance across all evaluation metrics. With a learning rate of 0.0001 and a batch size of 16, MobileNet reached an impressive accuracy of 99.44%, precision of 99.45%, recall of 99.44%, F1-score of 99.44%, and an exceptionally low log loss of 0.022, along with a near-perfect ROC-AUC score of 0.9993. These results confirm its robust predictive capability and strong reliability. Similarly, Xception and DenseNet201 also performed remarkably well, achieving accuracies of 98.74% and 98.73%, respectively, under optimal conditions. These models demonstrated high precision, recall, and F1-scores, supported by ROC-AUC scores exceeding 0.998. The findings underscore the potential of advanced deep learning architectures, particularly MobileNet, as reliable tools for automating the classification of MTM-MC relationships. This automation not only minimizes inter-observer variability but also enhances diagnostic consistency, paving the way for improved clinical decision-making and surgical outcomes. Future research should explore integrating these models into clinical workflows, focusing on their application in diverse patient populations and varying clinical scenarios.

## Data Availability

The datasets generated and analysed during this study are available from the corresponding author upon reasonable request. The ethic for this study was given by Necmettin Erbakan University Ethics Committee with the Decision Number: 2024/388 (Application ID: 18,250.R1).
